# Extreme phenotype-derived machine learning reveals susceptibility and resilience signatures for severe diabetic retinopathy

**DOI:** 10.3389/fendo.2026.1933878

**Published:** 2026-08-27

**Authors:** Jing-Yi Zhang, Yue-Ming Shu, Fang-Yuan Yang, Si-Wei Cai, Rong-Rong Xie, Ran Sun, Wei Tian, Xiao-Rong Zhu

**Affiliations:** 1 Department of Endocrinology, Beijing Diabetes Institute, Beijing Tongren Hospital, Capital Medical University, Beijing, China; 2 Beijing Diabetes Institute,Beijing Tongren Hospital, Capital Medical University, Beijing, China; 3 Department of Ophthalmology, Beijing Tongren Hospital, Capital Medical University, Beijing, China; 4 Outpatient Department, Beijing Tongren Hospital, Capital Medical University, Beijing, China; 5 Healthy Management Center, Beijing Tongren Hospital, Capital Medical University, Beijing, China

**Keywords:** diabetic retinopathy, extreme phenotype, LightGBM, machine learning, resilience, SHAP, susceptibility

## Abstract

**Background:**

The mechanisms underlying why some individuals with diabetes develop severe diabetic retinopathy (DR), whereas others remain free of retinal complications despite long-standing disease, remain incompletely understood. We aimed to identify systemic susceptibility and resilience signatures associated with severe DR using an extreme phenotype-derived machine learning framework.

**Methods:**

An extreme phenotype cohort was established comprising 712 individuals with diabetes mellitus, including 437 patients with proliferative diabetic retinopathy (PDR; susceptible phenotype) and 275 patients with diabetes duration ≥10 years without retinopathy (resilient phenotype). Clinical and biochemical variables were integrated to develop interpretable machine learning models. The optimal model was further interpreted using SHapley Additive exPlanations (SHAP). An independent community-based diabetic cohort (n=673) was used to evaluate the distribution of susceptibility signatures in real-world populations.

**Results:**

LASSO regression identified 21 phenotype-associated features for model development. Among the evaluated algorithms, LightGBM demonstrated the strongest ability to discriminate susceptible and resilient phenotypes, achieving an area under the receiver operating characteristic curve (AUC) of 0.90 (95% CI, 0.84–0.95) in the internal validation cohort. SHAP analysis identified urinary albumin excretion rate (UAER), diabetes duration, serum creatinine, total protein, age, and hypertension duration as the dominant phenotype-defining features. Notably, UAER exhibited a pronounced nonlinear association with susceptibility scores, suggesting a close link between renal microvascular injury and vulnerability to severe DR. When applied to the community cohort, the susceptibility signature showed limited discrimination in the overall population (AUC = 0.54) but became progressively enriched among individuals with greater metabolic burden, reaching an AUC of 0.71 in participants with fasting blood glucose ≥9.0 mmol/L.

**Conclusion:**

Using an extreme phenotype-derived machine learning framework, we identified systemic susceptibility and resilience signatures associated with severe diabetic retinopathy. Renal dysfunction, albuminuria, glycemic burden, and disease duration emerged as key phenotype-defining characteristics. These signatures became increasingly enriched in metabolically stressed individuals, supporting the concept that severe diabetic retinopathy arises through the interaction between intrinsic biological susceptibility and cumulative metabolic exposure. This framework may provide new insights into disease heterogeneity and facilitate future precision risk stratification strategies in diabetic eye disease.

## Introduction

Diabetic retinopathy (DR) remains a leading cause of preventable blindness among working-age adults globally (1). Its advanced stage, proliferative diabetic retinopathy (PDR), is characterized by retinal neovascularization and carries an imminent risk of severe, irreversible vision loss (2). Given the irreversible nature of PDR-induced structural damage, early identification and timely intervention for high-risk patients are paramount to preserving visual function (3).

Although chronic hyperglycemia, diabetes duration, hypertension, and renal dysfunction have been consistently associated with DR progression, considerable inter-individual heterogeneity exists in clinical practice (4–6). Some patients develop advanced retinopathy after relatively short periods of diabetes exposure, whereas others remain completely free of retinal lesions despite decades of disease (7). This phenomenon suggests that severe DR development is not solely determined by cumulative metabolic burden but may also reflect intrinsic differences in biological susceptibility and resilience to microvascular injury (8, 9). Understanding these mechanisms may provide important insights into disease pathogenesis and facilitate more precise risk stratification (9). However, the systemic characteristics distinguishing highly susceptible individuals from those exhibiting long-term resistance to retinal damage remain incompletely understood.

Most previous machine learning studies have focused on developing predictive models for DR detection using heterogeneous diabetic populations (10, 11). While these approaches have demonstrated promising discriminative performance, they are often limited by substantial phenotypic overlap between cases and controls (12). In particular, individuals classified as non-diabetic retinopathy (NDR) frequently include patients with relatively short diabetes duration who may subsequently develop retinal lesions during follow-up (4, 13). Such heterogeneity can obscure biologically meaningful signals and reduce the ability to identify mechanisms underlying disease susceptibility (14). Moreover, models trained in mixed populations primarily optimize prediction accuracy rather than revealing the biological factors that differentiate vulnerable from protected individuals (15).

Extreme phenotype designs provide a powerful strategy for overcoming this limitation (14). By comparing individuals located at opposite ends of the disease spectrum, biological contrast can be maximized and disease-associated signals amplified (16). This approach has been successfully applied in genetic and complex disease research to identify factors associated with disease susceptibility and resistance. In the context of DR, patients with proliferative retinopathy represent an extreme susceptible phenotype, whereas individuals who remain retinopathy-free despite long-standing diabetes constitute a unique resilient phenotype (9). Direct comparison between these two groups offers a valuable opportunity to investigate systemic determinants associated with severe retinal microvascular injury (17).

Recent advances in artificial intelligence (AI) have expanded the application of computational approaches in biomedical research and clinical decision-making. Deep learning architectures, including convolutional neural networks (CNNs) and transformer-based models, have demonstrated the ability to extract complex patterns from high-dimensional medical data and improve disease recognition. Recent studies, including DCNNBT, Tri-STANet, HEAL-LLMF, and XAI-RACapsNet, have highlighted the potential of deep learning, transformer-based architectures, multimodal learning, and explainable AI frameworks for medical data analysis and clinical decision support (18–21). In addition, machine learning approaches have explored strategies to improve model robustness under challenging conditions such as class imbalance, further expanding their applications in biomedical prediction tasks (22). However, most existing AI applications in healthcare, including DR research, have primarily focused on image-based diagnosis, screening, or prediction tasks. Models based on clinical variables remain comparatively underexplored for identifying the systemic signatures that characterize individuals with extreme susceptibility or resilience to severe DR. Interpretable machine learning approaches provide a promising solution by capturing nonlinear relationships among routinely available clinical features while allowing investigation of the contribution of individual predictors (23). In particular, tree-based ensemble algorithms such as Light Gradient Boosting Machine (LightGBM) can effectively model high-order interactions among metabolic, cardiovascular, and renal biomarkers (24, 25). When combined with interpretable artificial intelligence frameworks such as SHapley Additive exPlanations (SHAP), these approaches enable not only discrimination between phenotypic groups but also identification of the clinical features that contribute most strongly to disease susceptibility or resilience (26). This interpretability is especially important for translating computational findings into clinically meaningful biological insights.

Therefore, the primary objective of this study was not to develop a novel machine learning algorithm or a universal screening model for DR, but rather to apply an extreme phenotype framework to identify systemic susceptibility and resilience signatures associated with severe diabetic retinopathy. We constructed an interpretable machine learning model by contrasting patients with proliferative diabetic retinopathy against long-term diabetes patients who remained free of retinopathy. Furthermore, we evaluated the presence and distribution of these extreme phenotype-derived signatures in an independent community-based diabetic cohort to explore their translational relevance in real-world populations. Through this strategy, we sought to better understand the biological characteristics that distinguish vulnerable from protected individuals and to provide a foundation for future precision risk stratification in diabetic retinopathy.

## Methods

### Study design and participants

To identify systemic signatures associated with susceptibility and resilience to severe diabetic retinopathy (DR), we established an extreme phenotype cohort consisting of individuals located at opposite ends of the diabetic retinal disease spectrum (14). The rationale underlying this design was to maximize biological contrast and facilitate the identification of clinical and biochemical features associated with retinal microvascular vulnerability or protection.

The discovery cohort comprised 712 patients with type 2 diabetes mellitus (T2DM) who were admitted to Beijing Tongren Hospital between 01/01/2015 and 31/12/2017. Patients with type 1 diabetes mellitus were excluded to minimize heterogeneity arising from differences in disease pathophysiology, metabolic characteristics, and retinopathy progression patterns. Participants were categorized into two extreme phenotypic groups. The susceptible phenotype group included 437 patients diagnosed with proliferative diabetic retinopathy (PDR), confirmed by fundus fluorescein angiography and independent ophthalmologic evaluation. The resilient phenotype group consisted of 275 individuals with a documented diabetes duration of at least 10 years who remained free of detectable diabetic retinopathy based on standardized retinal screening. Eye phenotype screening was performed between April 2015 and July 2017 at Beijing Tongren Hospital, China. Digital retinal photographs of both eyes (2 eyes × 2 fields) were obtained using a TRC NW7SF non-mydriatic retinal camera (Topcon Corporation, Tokyo, Japan) at a 45°field of view. The photographs were independently evaluated by two qualified retinal graders according to standardized quality assurance protocols. The severity of diabetic retinopathy was graded according to the International Clinical Diabetic Retinopathy and Diabetic Macular Edema Disease Severity Scale. Exclusion criteria included severe non-diabetic ocular disorders, active infection, severe trauma, malignancy, and variables with more than 30% missingness. Variables exceeding this threshold were excluded because excessive missing information may introduce substantial uncertainty and reduce the reliability of subsequent machine learning analyses. This retrospective study adhered to the principles of the Declaration of Helsinki and was approved by the Institutional Review Board of Beijing Tongren Hospital, Capital Medical University (TREC2023-KY059).

To evaluate the translational relevance of the identified susceptibility signatures, an independent community-based cohort comprising 673 individuals with type 2 DM was recruited between 01/01/2023 and 31/12/2024. This cohort represented a real-world diabetic population with varying levels of metabolic control and disease severity.

### Data collection and preprocessing

A total of 47 baseline clinical and biochemical features were extracted from the electronic medical record system. After excluding variables with a missingness rate > 30%, 27 routine features were retained. To address multicollinearity, which can compromise model performance, we assessed inter-feature dependencies using Spearman’s rank correlation analysis. When two features exhibited a correlation coefficient exceeding 0.9, the variable showing a weaker association with the outcome was systematically removed. These encompassed demographics and clinical characteristics (sex, age, smoking, drinking, hypertension, duration of hypertension, duration of diabetes, body mass index (BMI), systolic blood pressure (SBP), and diastolic blood pressure (DBP)), glucose and lipid metabolic indicators (lipoprotein(a) (LPA), triglycerides (TG), total cholesterol (TC), low-density lipoprotein cholesterol (LDL), high-density lipoprotein cholesterol (HDL), fasting blood glucose (FBG), and glycated hemoglobin (HbA1c)), and liver and kidney function parameters (blood urea nitrogen (BUN), serum creatinine (Scr), uric acid (UA), total protein (TP), albumin (ALB), total bilirubin (TBIL), direct bilirubin (DBIL), alanine aminotransferase (ALT), aspartate aminotransferase (AST), and urinary albumin excretion rate (UAER)). For variables with missing data, a K-Nearest Neighbors (KNN) imputation algorithm (K = 5) was deployed to preserve latent physiological correlations and maximize statistical power (27). Subsequently, all continuous variables were standardized using Z-score transformation prior to downstream modeling.

### Identification of phenotype-associated features and machine learning modeling

To minimize the risk of overfitting and avoid potential information leakage during feature selection, the extreme discovery cohort was first randomly partitioned into a training set (80%) and an internal validation set (20%) using stratified sampling to preserve the distribution of PDR and resilient phenotypes. Feature selection was subsequently performed exclusively within the training cohort. The Least Absolute Shrinkage and Selection Operator (LASSO) logistic regression model with L1 regularization was applied to identify the most informative predictors from the candidate variables (28). A 10-fold stratified cross-validation procedure was used within the training cohort to determine the optimal penalization parameter (λ) based on the minimum binomial deviance. By shrinking the coefficients of less informative variables toward zero, LASSO reduced model complexity and selected 21 predictors with non-zero coefficients for subsequent machine learning model development.

Five supervised machine learning algorithms, including Logistic Regression (LR), Random Forest (RF), Support Vector Machine (SVM), eXtreme Gradient Boosting (XGBoost), and LightGBM, were subsequently trained using the selected features in the training cohort. No variables related to ophthalmologic diagnosis, retinal imaging findings, or criteria used for defining PDR status were included as predictors to prevent potential label leakage. Because the phenotype groups showed moderate imbalance, class weights were applied during model training rather than synthetic oversampling approaches. Model performance was then evaluated in the independent internal validation cohort.

### Model interpretation and identification of susceptibility–resilience signatures

To improve interpretability and identify the clinical features underlying susceptibility and resilience phenotypes, SHAP analysis was performed on the best-performing model. SHAP values quantify the contribution of each variable to the classification outcome at both individual and population levels (26). Variables with positive SHAP values were interpreted as contributing toward the susceptible phenotype, whereas negative SHAP values reflected characteristics associated with the resilient phenotype. Global SHAP importance rankings were generated to identify dominant phenotype-defining features. In addition, SHAP dependence plots were constructed to explore nonlinear associations and potential biological thresholds linking clinical variables with susceptibility to severe DR. This approach enabled the identification of clinically interpretable susceptibility and resilience signatures rather than relying solely on model discrimination metrics.

### Translation of extreme phenotype signatures to a community cohort

To assess whether susceptibility signatures identified in the extreme phenotype cohort remained detectable in real-world diabetic populations, the final LightGBM model was applied to the independent community cohort.

Because the community cohort represented a substantially broader spectrum of disease severity than the discovery cohort, analyses focused on evaluating the distribution and enrichment of susceptibility signatures rather than traditional external prediction performance alone. Predefined subgroup analyses were conducted according to clinically relevant metabolic and disease-duration thresholds. These analyses explored whether susceptibility signatures became increasingly enriched among individuals experiencing greater metabolic burden and prolonged diabetes exposure.

### Web deployment

**Figure 1:**
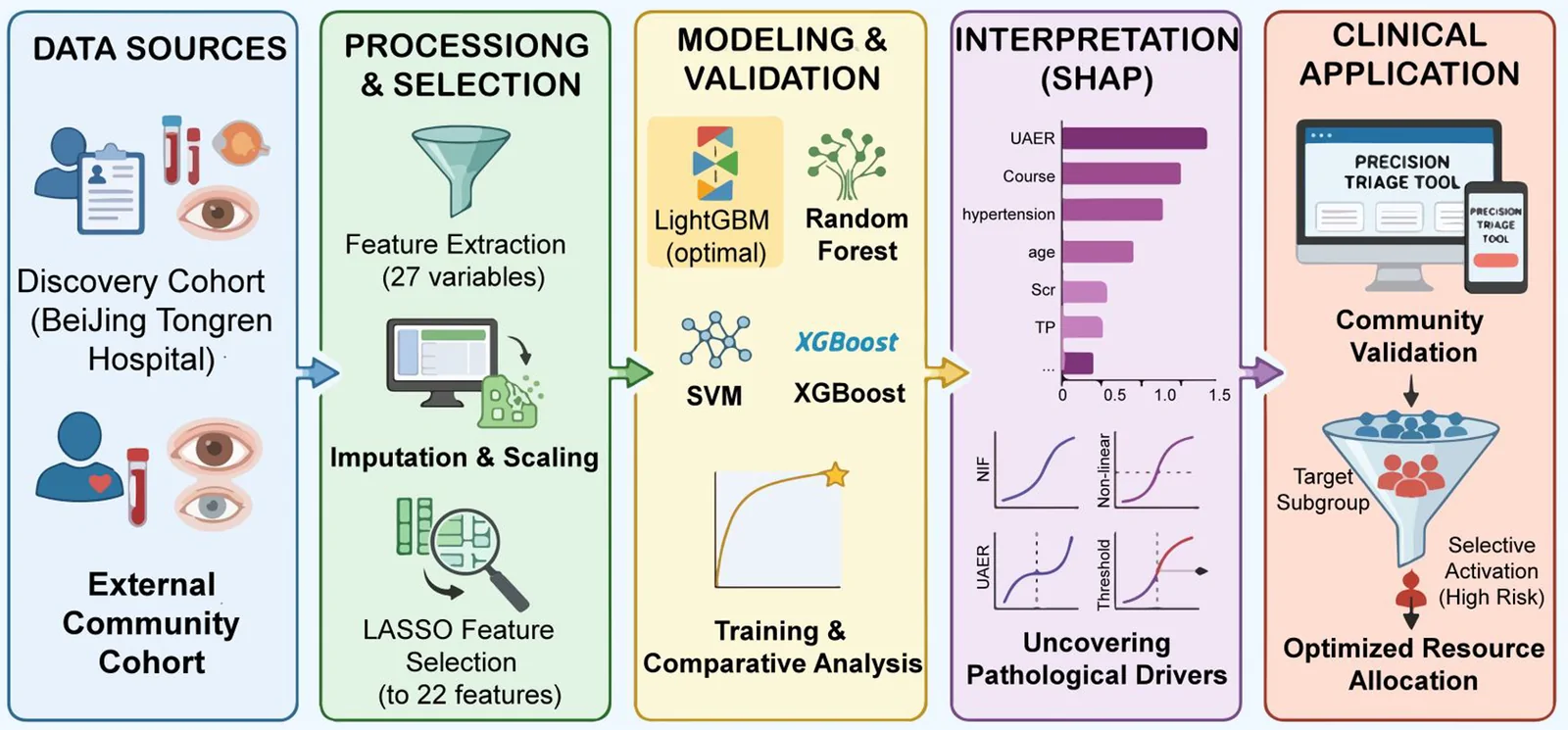
Study workflow for identifying susceptibility and resilience signatures associated with severe diabetic retinopathy using an extreme phenotype framework. LASSO, Least Absolute Shrinkage and Selection Operator; Light GBM, Light Gradient Boosting Machine; SVM, Support Vector Machine; XGBoost, Extreme Gradient Boosting; UAER, Urinary Albumin Excretion Rate; Scr, Serum Creatinine; TP, Total Protein.

To facilitate clinical translation, the final LightGBM model was implemented as an interactive web application using the Streamlit framework. Users can input routinely available clinical and biochemical variables through a structured interface. Rather than providing a simple disease probability estimate, the application generates an individualized susceptibility score reflecting the degree of similarity to the severe DR phenotype identified within the extreme phenotype cohort. The web-based platform was designed as a proof-of-concept decision support tool for biological risk stratification and targeted ophthalmologic surveillance. The comprehensive workflow of the study scheme is illustrated in **Figure 1**. The susceptibility score was defined as the predicted probability output from the LightGBM classifier, reflecting the model-estimated likelihood of an individual exhibiting the PDR-susceptible phenotype.

### Statistical analysis

Continuous variables were expressed as medians with interquartile ranges (IQR) and compared using the Mann-Whitney U test, as the data did not follow a normal distribution. Categorical variables were presented as frequencies and percentages (%), with differences compared using the Chi-square test or Fisher’s exact test. For machine learning models, discriminative performance was evaluated using the Area Under the Receiver Operating Characteristic Curve (AUC-ROC), with 95% confidence intervals (CIs) calculated via 1,000 bootstrap resamples. Optimal cut-offs were determined by the Youden Index. Additional metrics including Accuracy, Sensitivity, Specificity, F1-Score, and Average Precision (AP) were calculated. Decision Curve Analysis (DCA) was conducted to quantify net clinical benefit. All statistical analyses and machine learning modeling were performed using SPSS 13.0 (IBM, Chicago, IL, USA) and Python 3.12.3. A two-sided *P* < 0.05 was considered statistically significant.

## Results

### Baseline characteristics of the discovery cohort

The discovery cohort of this study included a total of 712 diabetic patients with extreme phenotypes, comprising 437 patients in the PDR group and 275 patients in the NDR group. Detailed demographic, clinical, and laboratory baseline characteristics of the two groups are summarized in **Table 1**.

**Table 1:** Demographic, clinical and laboratory data of the discovery cohort.

Parameters	PDR (N = 437)	NDR (N = 275)	*P* value
Male participants (%)	69.09	54.18	0.006*
Smoking (%)	27.23	32.36	0.022*
Drinking (%)	15.56	20.73	0.759
Hypertension (%)	61.56	54.55	0.452
Age (yr)	58.02 (56.47,59.57)	61.59 (60.17, 63.00)	<0.001*
Duration of Hypertension (yr)	5.05 (3.86, 6.26)	8.22 (6.79, 9.65)	0.005*
Duration of DM (yr)	14.58 (13.51, 15.64)	13.70 (13.11, 14.30)	0.552
BMI (kg/m^2^)	25.60 (25.01, 26.18)	26.26 (24.36, 28.16)	0.274
WHR	0.93 (0.92, 0.95)	0.93 (0.92, 0.94)	0.250
SBP (mmHg)	140.89 (138.06, 143.71)	132.48 (130.22, 134.73)	0.001*
DBP (mmHg)	83.37 (81.70, 85.03)	78.32 (76.90, 79.75)	0.007*
BUN (mmol/L)	5.72 (5.25, 6.18)	4.92 (4.70, 5.15)	<0.001*
Scr (μmol/L)	89.09 (81.55, 96.62)	73.20 (70.51, 75.90)	<0.001*
UA (μmol/L)	315.07 (303.71, 326.44)	320.87 (309.90, 331.83)	0.693
TP (g/L)	64.08 (63.11, 65.05)	64.05 (63.35, 64.76)	0.214
ALB (g/L)	36.13 (35.43, 36.83)	38.15 (37.59, 38.71)	<0.001*
TBIL (μmol/L)	14.93 (11.71, 18.16)	15.21 (14.42, 16.00)	0.003*
DBIL (μmol/L)	2.84 (2.57, 3.11)	4.74 (1.88, 7.60)	<0.001*
ALT (U/L)	20.43 (18.62, 22.23)	23.74 (21.56, 25.92)	0.003*
AST (U/L)	21.41 (20.19, 22.63)	23.09 (21.94, 24.23)	0.041*
TG (mmol/L)	2.17 (1.88, 2.47)	1.78 (1.64, 1.93)	0.097
TC (mmol/L)	5.08 (4.87, 5.25)	4.65 (4.52, 4.78)	0.001*
LDL (mmol/L)	3.20 (3.05, 3.35)	2.96 (2.85, 3.07)	0.027*
HDL (mmol/L)	1.22 (1.13, 1.31)	1.14 (1.10, 1.19)	0.714
FBG (mmol/L)	8.51 (7.95, 9.07)	7.31 (6.98, 7.64)	0.009*
HbA1c (%)	8.97 (8.66, 9.27)	8.32 (8.11, 8.54)	1.000
UAER (μg/min)	568.58 (384.37, 752.79)	35.45 (13.75, 57.15)	<0.001*

*P<0.05; PDR, Proliferative Diabetic retinopathy; NDR, No diabetic retinopathy; DM, Diabetes Mellitus; BMI, Body Mass Index; WHR, Waist- Hip Ratio; SBP, Systolic Blood Pressure; DBP, Diastolic Blood Pressure; BUN, Blood Urea Nitrogen; Scr, Serum Creatinine; UA, Uric Acid; TP, Total Protein; ALB, Albumin; TBIL, Total Bilirubin; DBIL, Direct Bilirubin; ALT, Alanine Aminotransferase; AST, Aspartate Aminotransferase; TG, Triglyceride; TC, Total Cholesterol; LDL, Low-Density Lipoprotein; HDL, High-Density Lipoprotein; FBG, Fasting Blood Glucose; HbA1c, Glycated Hemoglobin; UAER, Urinary Albumin Excretion Rate.

Compared with the NDR group, PDR patients were more likely to be male (69.09% vs. 54.18%, *P* = 0.006) and were significantly younger (58.02 vs. 61.59 years, *P* < 0.001). Smoking prevalence was lower in the PDR group (27.23% vs. 32.36%, *P* = 0.022), whereas no significant differences were observed in drinking status or the prevalence of hypertension between the two groups. Hemodynamically, the PDR group exhibited significantly higher systolic and diastolic blood pressure (SBP: 140.89 vs. 132.48 mmHg; DBP: 83.37 vs. 78.32 mmHg; both *P* < 0.01), whereas hypertension duration was paradoxically shorter in PDR patients (5.05 vs. 8.22 years, *P* = 0.005), suggesting differences in disease trajectory rather than comorbidity burden.

In terms of metabolic and biochemical parameters, the PDR group demonstrated markedly elevated urinary albumin excretion rate (UAER) (568.58 vs. 35.45 μg/min, P < 0.001), indicating severe renal microvascular involvement. Consistently, renal function markers including blood urea nitrogen (BUN) and serum creatinine (Scr) were significantly higher in PDR patients.

Lipid metabolism was also significantly altered, with higher total cholesterol (TC) and low-density lipoprotein cholesterol (LDL) levels in the PDR group (both P = 0.001 and P = 0.027, respectively), suggesting a more adverse metabolic profile. Liver-related indices also showed significant alterations, including lower albumin (ALB) levels and changes in bilirubin and transaminases.

Notably, fasting blood glucose (FBG) was significantly higher in the PDR group (8.51 vs. 7.31 mmol/L, P = 0.009), whereas HbA1c did not differ between groups, likely reflecting long-term glycemic exposure saturation in this extreme phenotype design.

Overall, these findings indicate that severe diabetic retinopathy is associated with a multidimensional pattern of systemic metabolic, renal, and vascular abnormalities rather than a single dominant risk factor.

### Dimensionality reduction and optimal feature selection

**Figure 2:**
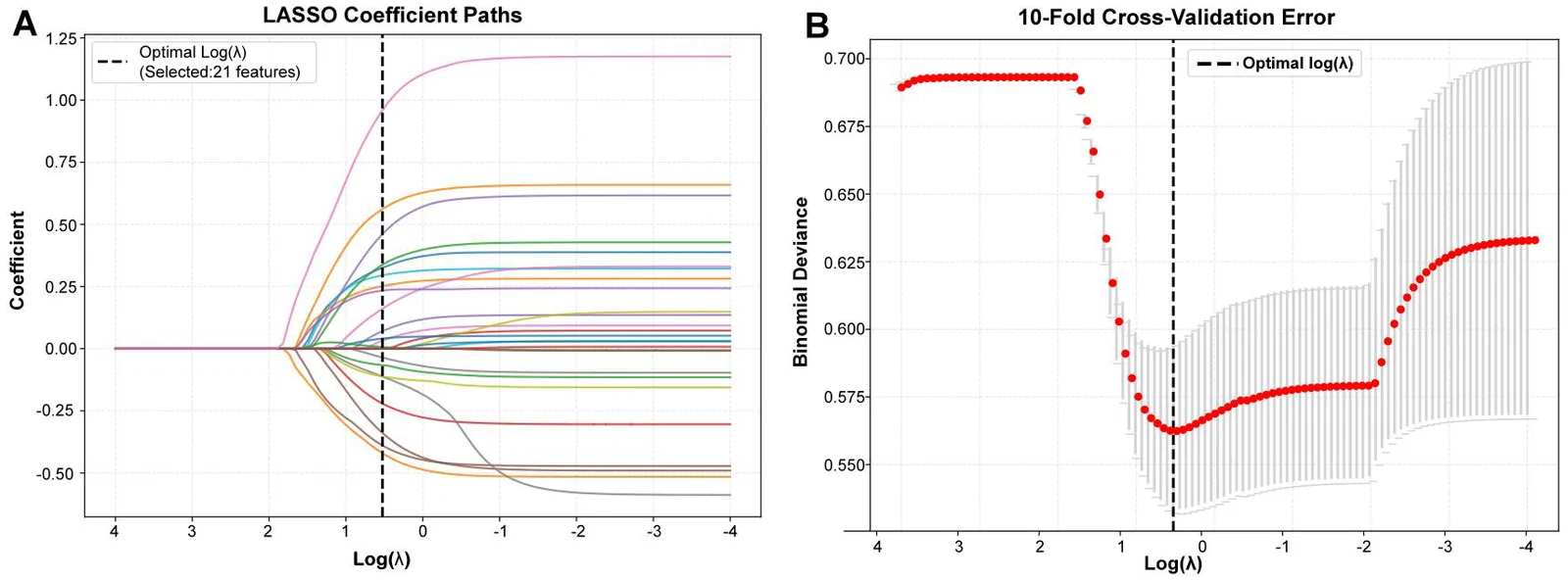
Feature selection and dimensionality reduction using the LASSO logistic regression model. **(A)** LASSO coefficient shrinkage paths of the candidate clinical and biochemical features. The x-axis represents the log-transformed penalization parameter log(λ), and the y-axis represents the coefficient values. Each colored curve illustrates the trajectory of an individual feature’s coefficient as the penalty weight changes. The vertical dashed line is drawn at the optimal log(λ) value, where exactly 21 non-zero core features were successfully retained. **(B)** 10-fold cross-validation error curve for tuning parameter log(λ) selection. The x-axis indicates log(λ), and the y-axis indicates the binomial deviance (cross-validation error). Red dots represent the mean binomial deviance across the 10 folds for each λ, with gray error bars indicating the standard error. The vertical dashed line highlights the optimal log(λ) that minimizes the cross-validation error, identifying the most robust predictive subset while avoiding overfitting. LASSO, Least Absolute Shrinkage and Selection Operator.

Following stratified partitioning of the discovery cohort into training and internal validation sets, LASSO logistic regression was performed exclusively within the training cohort. The coefficient shrinkage paths (**Figure 2A**) demonstrated that redundant variables systematically converged toward zero as the penalty intensity log(λ) increased. Following 10-fold cross-validation, at the optimal log(λ) threshold that minimized the binomial deviance (**Figure 2B**), the LASSO model successfully isolated 21 potential predictive features with non-zero coefficients. These indicators include: sex, age, smoking, hypertension, duration of hypertension, duration of diabetes, BMI, SBP, DBP, BUN, Scr, UA, TP, ALB, TBIL, DBIL, ALT, LDL, TC, FBG, and UAER. These retained features represented multiple biological domains, including glycemic control, renal function, blood pressure regulation, lipid metabolism, hepatic function, and demographic characteristics, suggesting that susceptibility and resilience to severe diabetic retinopathy arise from the interaction of multiple systemic pathways rather than a single dominant risk factor.

### Comprehensive performance of the five machine learning models

**Figure 3:**
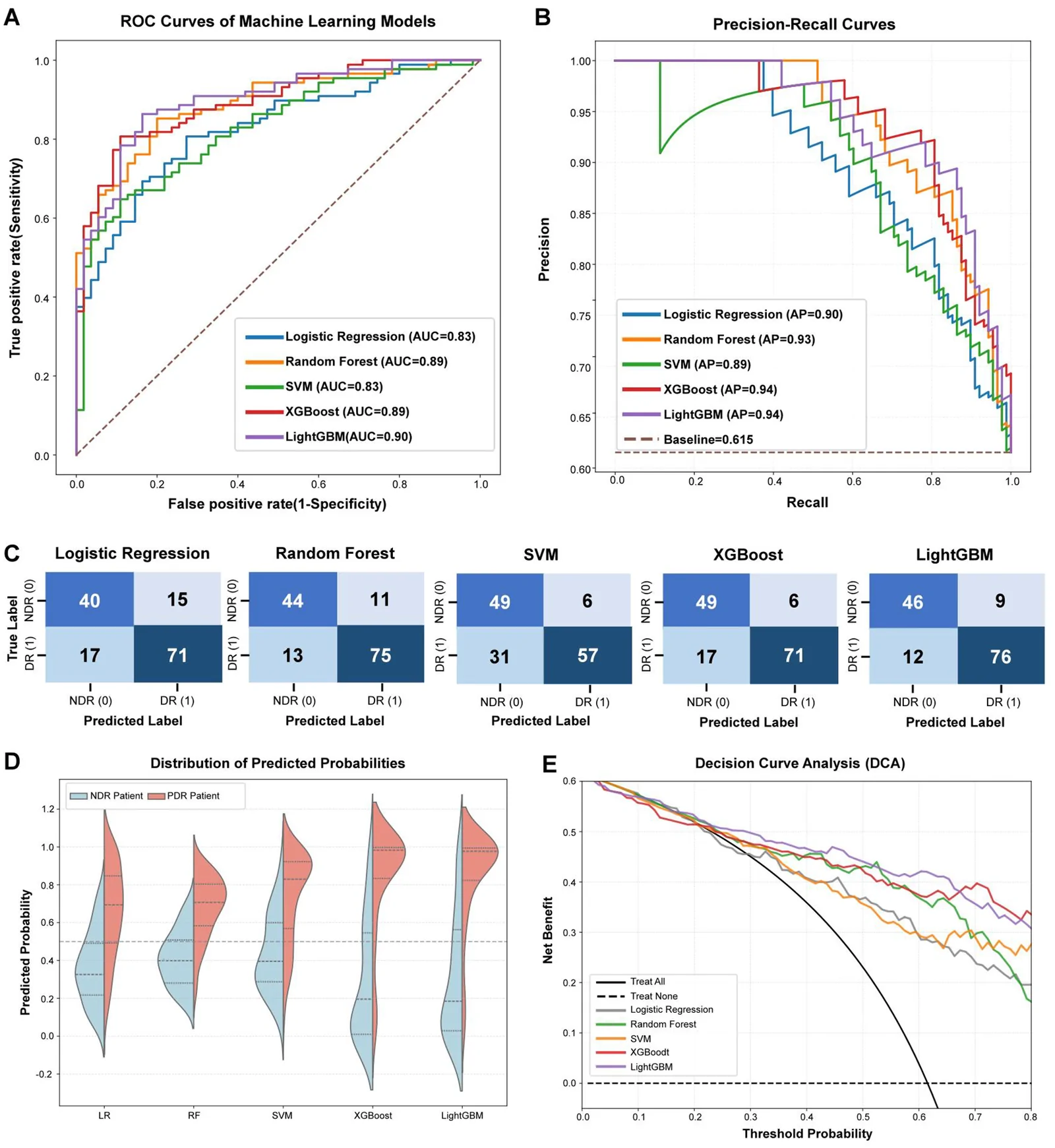
Comprehensive performance evaluation and clinical utility of the five machine learning models in the internal validation set. **(A)** ROC curves. Displays the trade-off between the true positive rate (sensitivity) and false positive rate (1 - specificity). The LightGBM model exhibited the highest discriminative ability with an Area Under the Curve (AUC) of 0.90. **(B)** Precision-Recall (PR) curves. Evaluates model performance with a focus on the correct identification of positive cases. The dashed line represents the baseline prevalence of DR in the dataset (0.62). Both Random Forest and LightGBM achieved the highest Average Precision (AP = 0.94). **(C)** Confusion matrices. Displays the absolute counts of true negatives, false positives, false negatives, and true positives based on the optimal Youden index cut-off values. This provides an intuitive visualization of the specific misclassification rates (e.g., missed diagnoses vs. overdiagnoses) for each algorithm. **(D)** Distribution of predicted probabilities. Split violin plots show the predicted probability scores generated by each model for true NDR patients (light blue) versus true DR patients (salmon). High-performing models (e.g., LightGBM) demonstrate distinct separation with minimal overlap between the two patient groups. **(E)** Decision Curve Analysis (DCA). Illustrates the clinical net benefit across a continuous range of threshold probabilities. The predictive models, particularly LightGBM and XGBoost, consistently provided a higher net benefit than the default strategies of “Treat All” (black solid line) and “Treat None” (black dashed line), confirming substantial clinical utility. ROC, Receiver Operating Characteristic; DR, Diabetic retinopathy; SVM, Support Vector Machine; XGBoost, Extreme Gradient Boosting; LightGBM, Light Gradient Boosting Machine.

Using the 21 core features selected by LASSO regression, we trained and internally validated five machine learning models. The detailed predictive metrics are summarized in **Table 2**. Overall, the tree-based ensemble models significantly outperformed the traditional linear and SVM models. Specifically, the LightGBM model exhibited the highest discriminative capacity with an AUC of 0.90 (95% CI: 0.84-0.95), closely followed by RF (AUC = 0.89) and XGBoost (AUC = 0.889) (**Figure 3A**). Furthermore, in the Precision-Recall analysis, both LightGBM and XGBoost achieved the highest AP score of 0.94, demonstrating remarkable robustness well above the baseline prevalence of 0.62 (**Figure 3B**). At the optimal cut-off threshold determined by the Youden index, LightGBM delivered the most balanced and clinically favorable classification performance, achieving an accuracy of 0.85 and the highest F1-Score of 0.88. Notably, LightGBM demonstrated an outstanding sensitivity of 0.86, indicating a minimal false negatives, which is important for identifying individuals with a susceptibility profile resembling severe DR (**Table 2**, **Figure 3C**). While SVM achieved the highest specificity (0.91), its sensitivity was unacceptably low (0.62). The split violin plots (**Figure 3D**) intuitively corroborate these findings, revealing that LightGBM and XGBoost most effectively polarized the predicted probabilities, with a dense concentration of NDR patients near 0 and DR patients near 1, displaying minimal overlap.

**Table 2:** Performance of five machine learning models for discriminating susceptibility and resilience phenotypes in the internal validation set.

Model	AUC (95% CI)	Accuracy	Sensitivity	Specificity	F1-Score
LR	0.83 (0.76-0.89)	0.78	0.81	0.73	0.82
RF	0.89 (0.83-0.94)	0.83	0.85	0.80	0.86
SVM	0.83 (0.76-0.90)	0.74	0.65	0.89	0.75
XGBoost	0.89 (0.84-0.95)	0.84	0.81	0.89	0.96
LightGBM	0.90 (0.84-0.95)	0.85	0.86	0.84	0.88

LR, Logistic Regression; RF, Random Forest; SVM, Support Vector Machine; XGBoost, Extreme Gradient Boosting; LightGBM, Light Gradient Boosting Machine.

To evaluate whether the models’ theoretical accuracy translates into actual clinical decision-making value, we performed Decision Curve Analysis (DCA) (**Figure 3E**). The DCA curves demonstrated that adopting LightGBM, XGBoost, or RF to guide DR interventions provided a substantially higher net clinical benefit compared to the default strategies of “Treat All” or “Treat None” across almost the entire range of threshold probabilities (from 0.05 to 0.80). Traditional Logistic Regression yielded the lowest net benefit among the evaluated models.

### Identification of susceptibility and resilience signatures by SHAP analysis

**Figure 4:**
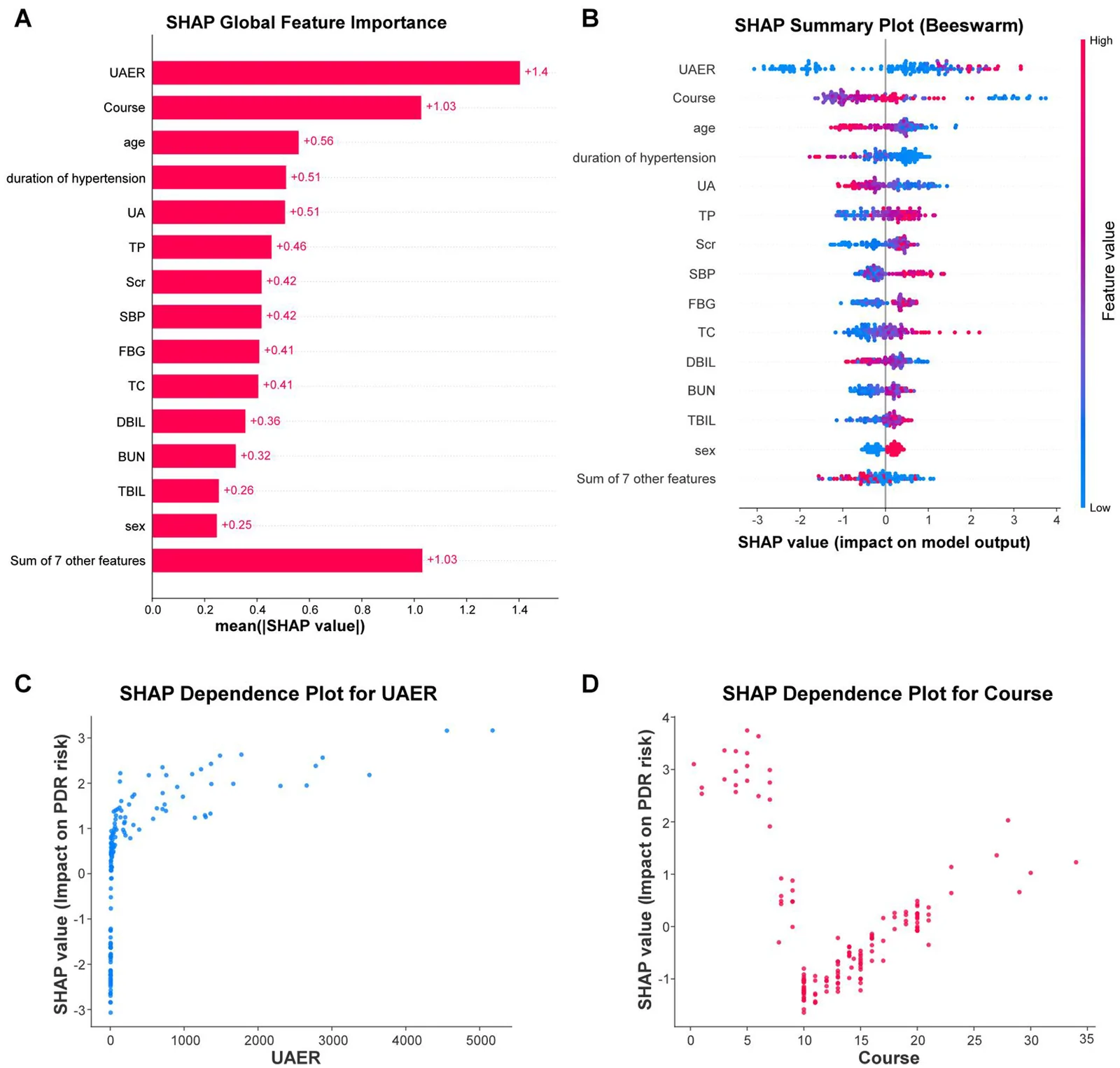
Identification of susceptibility and resilience signatures using SHAP analysis in the optimal LightGBM model. **(A)** Global SHAP importance ranking showing the relative contribution of each clinical feature to discrimination between susceptible and resilient phenotypes. **(B)** SHAP summary plot illustrating the direction and magnitude of feature effects. Positive SHAP values indicate greater similarity to the susceptible phenotype, whereas negative values indicate greater similarity to the resilient phenotype. **(C)** SHAP dependence plot for UAER. Demonstrates a pronounced nonlinear association between albuminuria and susceptibility scores. **(D)** SHAP dependence plot for diabetes duration. The elevated SHAP values observed at shorter diabetes durations reflect the extreme phenotype design, whereas susceptibility scores increase progressively beyond the resilient phenotype threshold. SHAP, SHapley Additive exPlanations; DR, Diabetic Retinopathy; NDR, No Diabetic Retinopathy; UAER, Urinary Albumin Excretion Rate; Scr, Serum Creatinine; TP, Total Protein; TC, Total Cholesterol; UA, Uric Acid; SBP, Systolic Blood Pressure; FBG, Fasting Blood Glucose; DBIL, Direct Bilirubin; BUN, Blood Urea Nitrogen; ALT, Alanine Aminotransferase.

To elucidate the biological features underlying phenotype discrimination, SHAP analysis was performed using the optimal LightGBM model (**Figure 4**). The global feature importance bar chart (**Figure 4A**) identified the UAER and the course of diabetes as the two most predominant predictors. The SHAP beeswarm plot (**Figure 4B**) further demonstrated that elevated UAER, prolonged diabetes duration, impaired renal function, and poorer glycemic control were generally associated with greater similarity to the susceptible phenotype, whereas lower values tended to characterize resilient individuals.

To delineate the fine-grained, non-linear effects of these top two indicators, SHAP dependence plots were constructed. As shown in **Figure 4C**, the influence of UAER on DR risk exhibited a stark threshold effect. The SHAP values spiked dramatically to a high positive level even with minor initial elevations in UAER, and subsequently plateaued. This specific pattern underscores that the onset of even early-stage microalbuminuria drastically amplifies the likelihood of severe retinopathy, functioning as a critical early-warning signal. A distinct nonlinear pattern was observed for diabetes duration (**Figure 4D**). The elevated SHAP values observed at shorter diabetes durations likely reflect the extreme phenotype design, whereby resilient individuals were required to remain free of retinopathy despite at least ten years of diabetes exposure. Beyond this threshold, susceptibility scores increased progressively with disease duration, consistent with cumulative metabolic injury contributing to severe DR development.

### External validation in the community-based cohort

**Figure 5:**
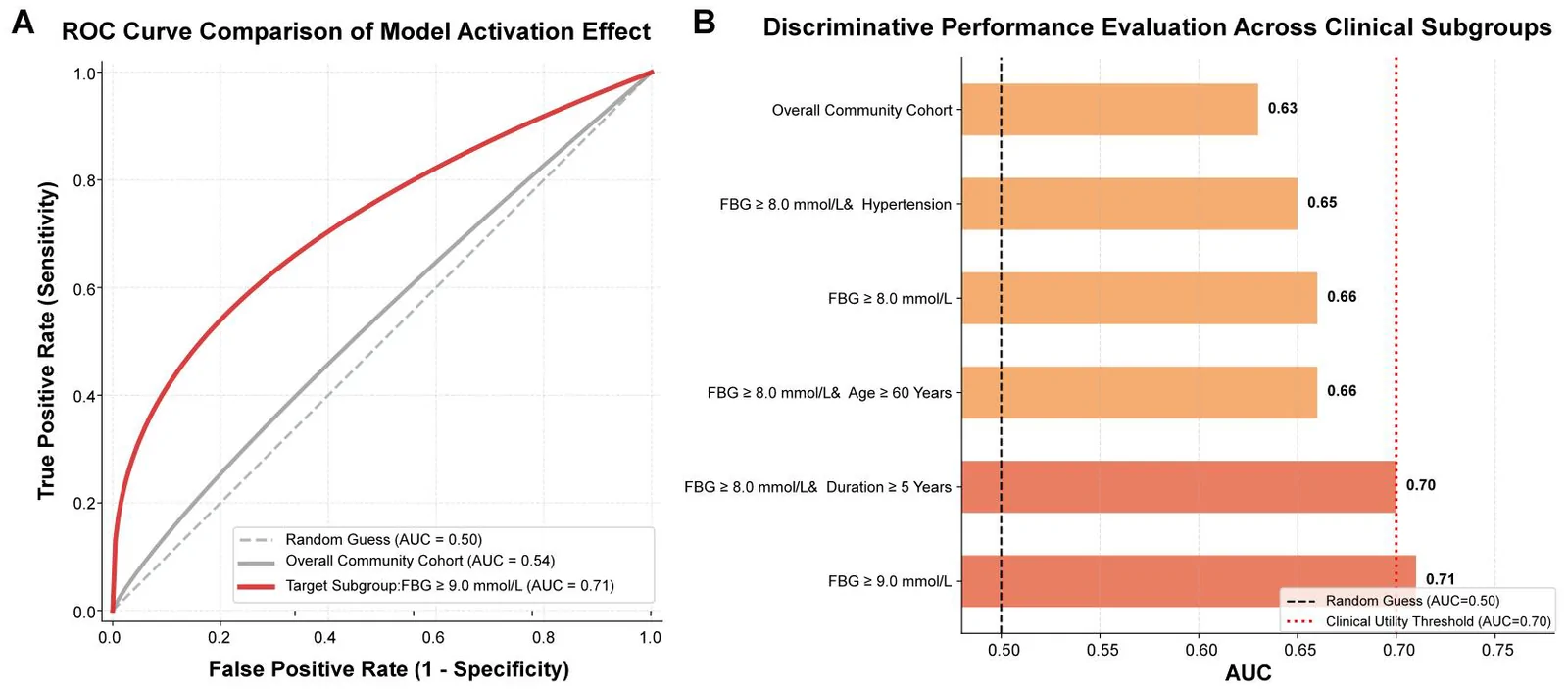
Distribution and enrichment of extreme phenotype-derived susceptibility signatures in an independent community-based diabetic cohort. **(A)** Receiver operating characteristic curves comparing the overall community cohort and the subgroup with fasting blood glucose ≥9.0 mmol/L. The susceptibility signature showed limited discrimination in the overall population but became more evident among individuals with greater metabolic burden. **(B)** Discriminative performance across predefined clinical subgroups. Progressive increases in AUC values were observed with increasing glycemic burden and disease duration, indicating enrichment of susceptibility-associated biological characteristics in metabolically stressed populations. ROC, Receiver Operating Characteristic; AUC, Area Under the Curve; FBG, Fasting Blood Glucose.

The susceptibility signature identified in the extreme phenotype cohort was subsequently evaluated in an independent community-based diabetic population. When applied to the entire community cohort, the model demonstrated limited discrimination (AUC = 0.55), indicating that the strong biological contrasts present in the extreme phenotype cohort became substantially diluted within a broader real-world population characterized by heterogeneous disease trajectories (**Figure 5A**).

To further explore the distribution of susceptibility signatures, predefined subgroup analyses were performed. Progressive enrichment of the susceptibility signature was observed with increasing metabolic burden. Model discrimination improved from an AUC of 0.55 in the overall community cohort to 0.70 among individuals with fasting blood glucose ≥8.0 mmol/L and diabetes duration ≥5 years, and further increased to 0.72 among individuals with fasting blood glucose ≥9.0 mmol/L (**Figure 5B**). These findings suggest that the biological signature identified through extreme phenotype analysis is not uniformly distributed across diabetic populations. Instead, it appears increasingly concentrated among individuals exposed to greater metabolic stress, supporting the concept that susceptibility to severe diabetic retinopathy emerges through the interaction between intrinsic vulnerability and cumulative metabolic burden.

### Clinical implementation and web-based interface

To facilitate translational application, the final LightGBM model was deployed as an interactive web-based platform. By entering routinely available clinical and biochemical variables, users can obtain an individualized susceptibility score reflecting the degree of similarity to the severe DR phenotype identified in the extreme phenotype cohort. The application is intended as a proof-of-concept tool for biological risk stratification and exploratory assessment of susceptibility signatures rather than direct disease diagnosis. The publicly accessible application is available at: https://8wvkwt6xq6zyc8ruk8kgut.streamlit.app/.

## Discussion

In this study, we employed an extreme phenotype framework combined with interpretable machine learning to investigate the systemic characteristics associated with severe DR. By contrasting patients with PDR, representing a highly susceptible phenotype, against long-term diabetes patients who remained completely free of retinopathy, representing a resilient phenotype, we identified a multidimensional clinical signature associated with vulnerability and protection against severe retinal microvascular injury. Among the evaluated algorithms, LightGBM achieved the strongest discrimination between the two phenotypes, while SHAP analysis revealed that renal dysfunction, glycemic burden, disease duration, and blood pressure-related variables were the dominant contributors to phenotype separation. Importantly, when translated into a community-based diabetic population, the identified susceptibility signature was not uniformly distributed but became progressively enriched among individuals with greater metabolic burden. Importantly, the conceptual contribution of this study lies primarily in the extreme phenotype framework, whereas machine learning served as an integrative analytical tool to capture multidimensional clinical patterns and enhance interpretability.

Most previous machine learning studies in DR have focused on disease prediction using heterogeneous diabetic populations, in which cases and controls often overlap substantially in clinical characteristics (10, 28). Such overlap may dilute biologically meaningful signals and limit the ability to identify determinants of disease susceptibility (14, 29). In contrast, extreme phenotype strategies maximize biological contrast by selecting individuals at opposite ends of the disease spectrum. Similar approaches have been widely used in genetic epidemiology and complex disease research to identify factors associated with disease susceptibility and resistance (16). In the present study, comparing PDR patients with long-term retinopathy-free individuals enabled the detection of systemic characteristics that may otherwise remain obscured within conventional case-control cohorts (9).

One of the most notable findings was the dominant contribution of uUAER to phenotype discrimination (13). UAER emerged as the most dominant discriminative feature, exhibiting an orders-of-magnitude difference between phenotypes and consistently ranking as the top contributor in SHAP analysis, highlighting its central role as a systemic marker of microvascular vulnerability. This observation supports the growing body of evidence suggesting that diabetic retinopathy and diabetic kidney disease share common microvascular mechanisms, including endothelial dysfunction, chronic inflammation, oxidative stress, and disruption of vascular barrier integrity (30). The strong association between albuminuria and the susceptible phenotype observed in our study reinforces the concept that retinal and renal microvascular injury may represent parallel manifestations of systemic vascular vulnerability rather than isolated organ-specific complications (31, 32).

Renal function markers, including serum creatinine and blood urea nitrogen, also contributed substantially to phenotype discrimination. Together with the prominence of UAER, these findings suggest that susceptibility to severe DR may reflect a broader systemic microvascular phenotype rather than pathology confined to the retina. Previous studies have demonstrated that renal dysfunction is associated with both the incidence and progression of diabetic retinopathy (33). Our findings extend this observation by suggesting that markers of renal injury are among the most important features distinguishing highly susceptible individuals from those who remain protected despite prolonged diabetes exposure (34).

The identification of a resilient phenotype is another important aspect of the present study. Although hyperglycemia is widely recognized as a major driver of diabetic complications, a subset of patients remain remarkably resistant to retinal injury despite long-standing diabetes (9). The biological basis of this resilience remains poorly understood (35). In our cohort, resilient individuals demonstrated substantially lower levels of albuminuria, better renal function, lower blood pressure, and more favorable metabolic profiles. These observations support the concept that protection against DR may not simply reflect the absence of risk factors but may instead represent active biological mechanisms that preserve microvascular integrity (35). Increasing attention has recently been directed toward disease resilience across multiple chronic diseases (9), and our findings suggest that resilience may represent an equally informative framework for understanding DR pathogenesis. Interestingly, individuals classified as the susceptible phenotype were younger than those in the resilient phenotype group. This finding should be interpreted in the context of the extreme phenotype design. The younger age observed in the susceptible phenotype likely reflects the enrichment of individuals who developed severe retinopathy at an earlier stage of life, rather than a protective effect of aging itself. Conversely, resilient individuals were required to remain free of retinopathy despite prolonged diabetes exposure, resulting in an inherently older age distribution. Therefore, age in this setting should be viewed primarily as a marker of phenotype selection rather than an independent protective factor.

Another noteworthy finding was that diabetes duration was not significantly different between the susceptible and resilient phenotypes in traditional univariate comparisons, yet ranked among the most influential variables in the SHAP analysis (26). This discrepancy highlights a key advantage of machine learning-based approaches over conventional statistical methods. Rather than relying solely on average group differences, machine learning models can capture nonlinear effects and complex interactions among clinical variables (26). The observed SHAP pattern suggests that diabetes duration alone may not determine susceptibility to severe diabetic retinopathy; instead, its impact appears to depend on the broader metabolic and microvascular context. This finding supports the concept that severe diabetic retinopathy results from the interplay between cumulative exposure and intrinsic biological susceptibility rather than duration alone (36).

An important observation emerged from the community-based validation analyses. When applied to the overall community cohort, the susceptibility signature exhibited limited discriminatory performance (17). Rather than indicating failure of the identified signature, this finding likely reflects the biological heterogeneity of real-world diabetic populations. The discovery cohort was intentionally enriched for extreme phenotypes, whereas community populations contain individuals spanning a continuous spectrum of susceptibility and disease severity (14). Consequently, the strong biological contrasts present in the extreme phenotype cohort are expected to become attenuated when applied to a broader population.

Notably, the susceptibility signature became progressively enriched among individuals experiencing greater metabolic burden, particularly those with poor glycemic control and longer disease duration (13). These findings suggest that severe DR development may arise through the interaction of two complementary processes: intrinsic susceptibility and cumulative metabolic exposure. In this framework, susceptibility-related biological characteristics may remain clinically silent in early disease stages but become increasingly apparent as metabolic stress accumulates. This observation may help explain why some patients develop severe retinopathy despite relatively modest diabetes duration, whereas others remain protected even after prolonged exposure (35).

The present findings should be interpreted within the context of several limitations. First, the study employed a retrospective design, and causal relationships cannot be established. Second, the extreme phenotype strategy intentionally maximizes biological contrast and therefore may not fully represent the broader diabetic population (29). Third, all variables were derived from routinely collected clinical data, and molecular mechanisms underlying susceptibility and resilience could not be directly evaluated. Future studies incorporating genomics, proteomics, metabolomics, and longitudinal follow-up will be necessary to elucidate the biological pathways responsible for the observed phenotypic differences. Finally, although an independent community cohort was used for evaluation, the reduced discrimination observed in this setting indicates that the extreme phenotype-derived signature does not directly translate into a population-wide prediction model. Larger prospective cohorts with standardized phenotyping will be required to determine how these signatures can be adapted for broader clinical risk stratification. Although several identified features overlap with established risk factors for diabetic retinopathy, our extreme phenotype framework provides a complementary approach by capturing the combined clinical characteristics associated with susceptibility and resilience under prolonged diabetic exposure.

In conclusion, using an extreme phenotype-derived machine learning framework, we identified systemic susceptibility and resilience signatures associated with severe diabetic retinopathy. Renal dysfunction, albuminuria, glycemic burden, and disease duration emerged as major phenotype-defining characteristics. These signatures remained detectable within community populations and became increasingly enriched among individuals exposed to greater metabolic stress. Our findings suggest that severe diabetic retinopathy may result from the interaction between intrinsic biological susceptibility and cumulative metabolic burden, providing a potential framework for future biological stratification and precision screening strategies in diabetic eye disease.

## Data Availability

The original contributions presented in the study are included in the article/**Supplementary Material**. Further inquiries can be directed to the corresponding authors.

## Supplementary material

Supplementary Figure 1 Bubble plot of Spearman correlation analysis among various variables. PDR, Proliferative Diabetic retinopathy; BMI, Body Mass Index; WHR, Waist- Hip Ratio; SBP, Systolic Blood Pressure; DBP, Diastolic Blood Pressure; BUN, Blood Urea Nitrogen; Scr, Serum Creatinine; UA, Uric Acid; TP, Total Protein; ALB, Albumin; TBIL, Total Bilirubin; DBIL, Direct Bilirubin; ALT, Alanine Aminotransferase; AST, Aspartate Aminotransferase; TG, Triglyceride; TC, Total Cholesterol; LDL, Low-Density Lipoprotein; HDL, High-Density Lipoprotein; FBG, Fasting Blood Glucose; HbA1c, Glycated Hemoglobin; UAER, Urinary Albumin Excretion Rate.
