# Supplementary material for: Extreme phenotype-derived machine learning reveals susceptibility and resilience signatures for severe diabetic retinopathy
Source: Front Endocrinol (Lausanne). 2026 Aug 27;17:1933878. doi: 10.3389/fendo.2026.1933878 (PMC13557971; doi:10.3389/fendo.2026.1933878)
Supplement: Supplementary file 1 [file Supplementaryfile1.docx]

**Supplementary Figure 1. Bubble plot of Spearman correlation analysis among various variables.** PDR: Proliferative Diabetic retinopathy; BMI: Body Mass Index; WHR: Waist- Hip Ratio; SBP: Systolic Blood Pressure; DBP: Diastolic Blood Pressure; BUN: Blood Urea Nitrogen; Scr: Serum Creatinine; UA: Uric Acid; TP: Total Protein; ALB: Albumin; TBIL: Total Bilirubin; DBIL: Direct Bilirubin; ALT: Alanine Aminotransferase; AST: Aspartate Aminotransferase; TG: Triglyceride; TC: Total Cholesterol; LDL: Low-Density Lipoprotein; HDL: High-Density Lipoprotein; FBG: Fasting Blood Glucose; HbA1c: Glycated Hemoglobin; UAER: Urinary Albumin Excretion Rate.
